# Introduction of ligated vessels promote the retention and regeneration of free fat: constructing a fat flap in tissue engineering chamber

**DOI:** 10.1080/21623945.2020.1735025

**Published:** 2020-03-03

**Authors:** Chen Lei, Beichen Cai, Xiaobin Chen, Zhiyong Huang, Biao Wang

**Affiliations:** Department of Plastic and Cosmetic Surgery, The First Affiliated Hospital of Fujian Medical University, Fuzhou, Fujian, P.R. China

**Keywords:** Fat graft, regeneration, tissue engineering chamber, tissue engineering, adipose tissue

## Abstract

**Background**: Breast reconstruction with fat grafting has an unstable retention rate due to insufficient revascularization. Tissue Engineering Chamber (TEC) model can promote tissue regeneration in the chamber by introducing ligated vessels around the tissue. We introduced ligated vessels with free fat graft to investigate the retention rate and revascularization of grafted fat that in TEC model.

**Methods**: SD rats (n=24) was divided into 3 groups randomly. Group A: Standard TEC model was constructed; Group B: the epigastric vessel bundles were dissected from the fat flap and ligated, fat flap was cut into granules and planted into the chamber; Group C: Free fat was planted in the chamber. At week 6, samples in the chamber were harvested.

**Results**: Significant volume increase was observed in group A and B, while the volume decreased in group C (P<0.05). Regeneration morphology could be found according to the histological observation in A and B. Micro CT results showed the ligated vessels into grafted fat sprouting robustly, coordinated with volume changes.

**Conclusion**: Fat grafts in TEC model could not only survive but also regenerate. The combination of fat graft and TEC could fabricate a vascularized fat flap, which was a promising method in breast reconstruction.

**Abbreviations:** VOI: Volumes of Interest; TEC: Tissue Engineering Chamber; CAL: Cell Assisted Lipotransfer.

## Introduction

In plastic surgery, there are tremendous clinical demands on correcting soft tissue defects or hypogenesis. Despite a variety of commercial products including synthetic implants, injectable filling material, and homologous or human-derived autografts, autologous fat transplantation is more commonly performed, due to its good tissue compatibility with minimal rejection reactions and donor site defects [1,2]. Since Neuber [1] first reported autologous fat transplantation, several technical and basic science landmark progress of autologous fat transplantation has been made including tumescent liposuction [1], structure lipotransfer [2], three zone theory [3,4], cell assisted lipotransfer (CAL) [5,6] and SVF-gel [7]. Nevertheless, the varying retention rate [8,9] after transplantation remains to be solved and when applied to large defects like breast reconstruction, a high number of grafting procedures are required [10]. Although there is no widely accepted or optimal procedure to achieve predictable results [11], early revascularization was considered as the key to achieve a successful fat graft and adipose-derived stromal cells were certificated to promote the early revascularization as the theoretical basement of cell assisted lipotransfer. In 2013, Kølle et al [12] achieved a more than 80% retention volume by adding cultured adipose-derived stem cells, despite previous literature demonstrating poor results from bolus injection secondary to central necrosis due to lack of revascularization [12].

Tissue engineering chamber (TEC) model was developed by Morrison et al [13] in 2003. It introduces bundles of vessels into tissue engineering construct, utilizing the sprouting angiogenesis of blood vessels under hypoxic conditions to revascularize the engineered construct. This model can promote the survival and proliferation of even myocardial cells [14]. A number of studies have shown their results of enlarging the volume of fat flaps in rats [15], rabbits [16] and pigs [17] in TEC. This technique was famous for its construction of 56.5 ml adipose tissue from its initial volume of 5 ml in a porcine model [17]. This kind of spontaneous regeneration seemed to be specific in adipose tissue. Here we introduced bundles of ligated vessels into free fat in a rigid silicone chamber, attempting to construct a fat flap in vivo. It is also a way to promote the revascularization of grafted fat in order to observe the survival mechanism of grafted fat.

## Methods

### Animals and study design

**Figure 1.**
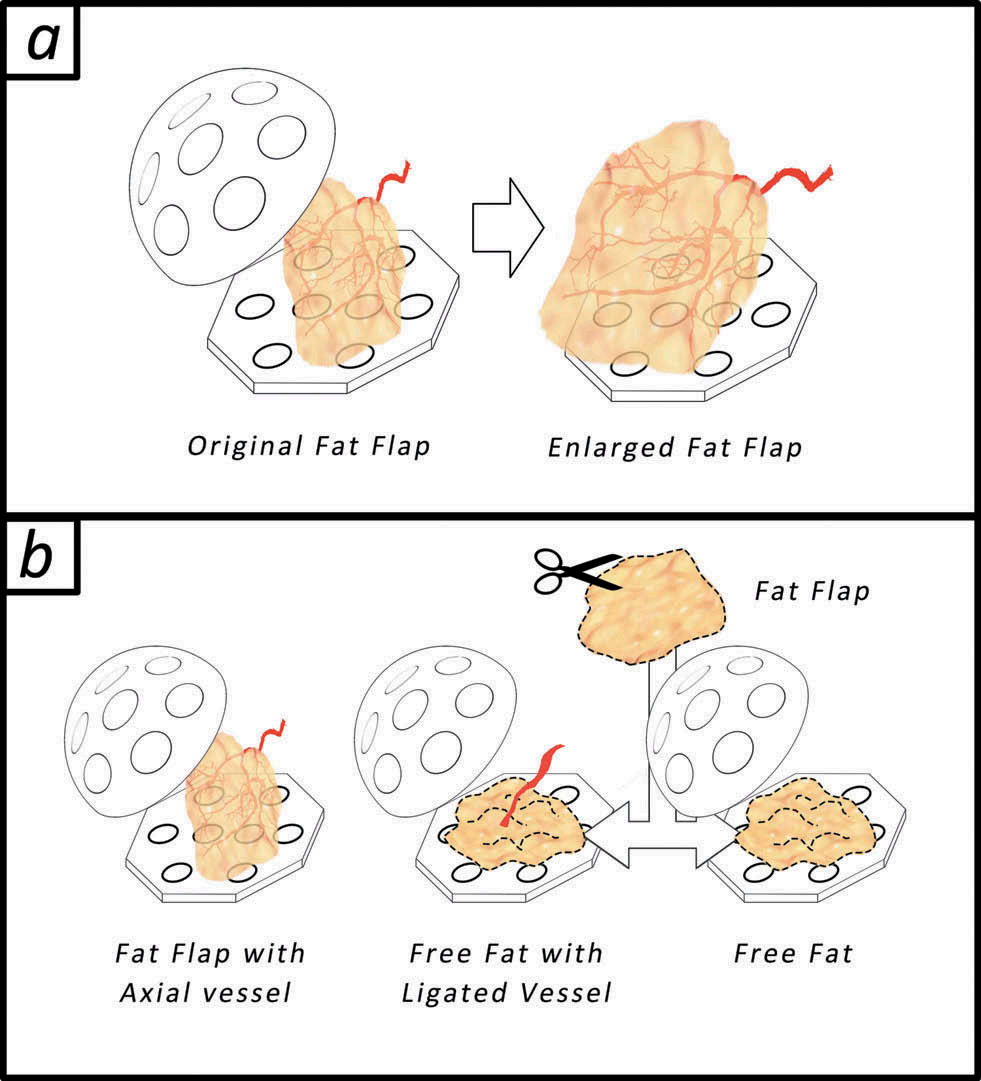
The graphic abstract. (a) tissue engineering chamber model for adipose tissue engineering, a fat flap (0.1 ml) with an axial vessel will regenerate after being transferred into the bigger rigid chamber (r = 1 cm, 2 ml). (b) Animals as divided in to 3 groups randomly: In group A: Standard tissue engineering chamber model was constructed; In group B: the epigastric vessel bundles was dissected from the fat flap and ligated, fat flap was cut into granules and planted into the chamber; In group (C) Free fat was planted in the chamber

All experiments were approved by Fujian Medical University Institutional Animal Care and use Committee. All animals were cared for in accordance with the Animal Welfare and Care of Fujian Medical University and Nation. Seven to nine-week-old male SD-rats weighing from 250–300 g were used in the experiments. All rats (n = 24) were divided into 3 groups as shown in Figure 1, the graphic abstract; Group A: Standard tissue engineering chamber model, the positive control, was constructed utilizing the epigastric vessel derived fat flap at the inguinal area as shown in Figure 1(b) left (n = 8); Group B: Instead of the fat flap, adipose tissue was cut into granules and planted into the silicone chamber with ligated epigastric vessel bundles as shown in Figure 1(b) middle (n = 8); Group C (n = 8): Free fat was planted in the chamber without extra vessels as shown in Figure 1(b), right.

### Surgical procedure

Before surgery, rats were anaesthetized using 10% chloral hydrate (0.3–0.4 mg/100 g) via intraperitoneal injection. A 3 cm incision was made after topical anaesthesia (2% lidocaine hydrocholoride injection) in the groin area. The target fat pad was right under the incision. After dissecting the soft tissue around, fat flap (0.1 ml) with vascular pedicle was isolated. Then a silicone hemisphere chamber (r = 1 cm, 2 ml) that was perforated with 2 mm holes was fixed on the abdominal wall next to the vessel pedicle using the 4–0 non-absorbable suture. In group A, the fat flap was transffered into the perforated tissue engineering chamber carefully so that the vascular pedicle was not pulled or twisted. In group B, the epigastric vessel bundles were dissected from the fat flap and ligated, the fat flap was cut into millimetre-sized granules and planted into the chamber as well. In group C, only fat granules were planted into the chamber. Then the wound was closed using interrupted horizontal mattress suture to prevent wound dehiscence. All the experimental animals were kept separately and looked sedated after surgery. Cefradine was used for three days after the operation to prevent infection.

### Tissue harvest and histological assessment

After 6 weeks, 5 rats in each group were anaesthetized again to harvest the grafted tissue along with the previous approach. We performed a clamp test to ensure that there was blood flow to refill the epigastric vessels. The volume of the tissue in the tissue engineering chamber was measured, and then the tissue was fixed in 4% paraformaldehyde, dehydrated and paraffin-embedded. Serial 7-μm paraffin sections were made parallel to the longitudinal axis (five slides in each sample at an average interval). Routine Haematoxylin and Eosin (H&E) staining was performed and then slides were assessed with light microscopy (Olympus BX51, Shinjuku ku, Japan) and phototographed (Olympus DP71, Shinjuku ku, Japan). The area with adipose tissue, connective tissue and necrosis (oil droplet more than 50 micron without cell structure) were measured with ImageJ software (National Institutes of Health, Bethesda, the United States) in H&E staining slides according to previous study [15]. Briefly, tissues were point counted 200 points and marked using image J from the five sections per construct. Samples were counted under high magnification so that the cell and tissue types could be readily identified. Vessels counts were

### Micro CT angiography

Three mice in each group were used to perform micro CT angiography. Microfil-122 [18] was used (Microfil ®, Flow Tech Inc, Carver, MA, the United States).Twenty-five ml of the contrast agent and 12 ml of diluent were mixed. The mixture was placed in a 37°C incubator. Before rats were euthanized, 0.9% saline containing heparin (100 U/ml) was injected intramuscularly for heparinization. The rats were fixed operating table, the chest and abdominal incision were made to expose the heart and liver. Then an 18 G catheter was inserted into the aorta. The catheter was fixed, the heparin saline was continuously perfused at the pressure of 115 mmHg. After the fluid flowing out from right atrium was clear and the liver was obviously whitened, 100 ml 10% formalin was used to perfuse and the rat body became stiff. While perfusing the formalin, about 1.4 ml of fixative was added to the pre-mixed contrast. After mixing, the contrast agent was injected from the aorta using a syringe. After the perfusion, the liver was filled with a yellow contrast agent. Then rats were placed in a refrigerator at 4°C overnight to wait for the fixation of the contrast agent. Micro-CT scans (u80, Scanco Medical) were performed at 36.0 μm voxel size at a voltage of 55 kVp and a current of 109 μA. A constant threshold of 220 was finally chosen to distinguish contrast agent from surrounding soft tissue and a low-pass Gaussian filter (σ = 1.2, support = 1) was used to suppress noise. Then initialize the micro-CT scanning to evaluate the perfused vessels and fat flaps. All the fat flaps including their perfused vessels were defined as volumes of interest (VOI). Vascular structure was computed by self-contained software. The results present as reconstructive 3D-graphs.

### Statistical analysis

All count data were expressed as mean ± SD. Statistical analyses were carried out with GraphPad Prism 7.0 software. Comparison between groups was performed by random data two-way ANOVA. Significance was established for p values of at least <0.05.

## Results

### Macroscopic observation and volume measurement

All animals recovered well after surgery. No infections or other complications happened. When harvesting at week 6, there was a layer of an connective tissue capsule enveloping the tissue engineering chamber in all groups. After peeling off the connective tissue capsule, There was exudate-like fluid flowing out of the chamber as described in previous studies [19,20]. Fibrous tissue invaded into the chamber from the side hole of the engineering chamber and grew with the tissue within the chamber as a whole. The tissue within the chamber was also wrapped by the fibrous tissue. No obvious oil cyst was observed in any of the 3 groups.

**Figure 2.**
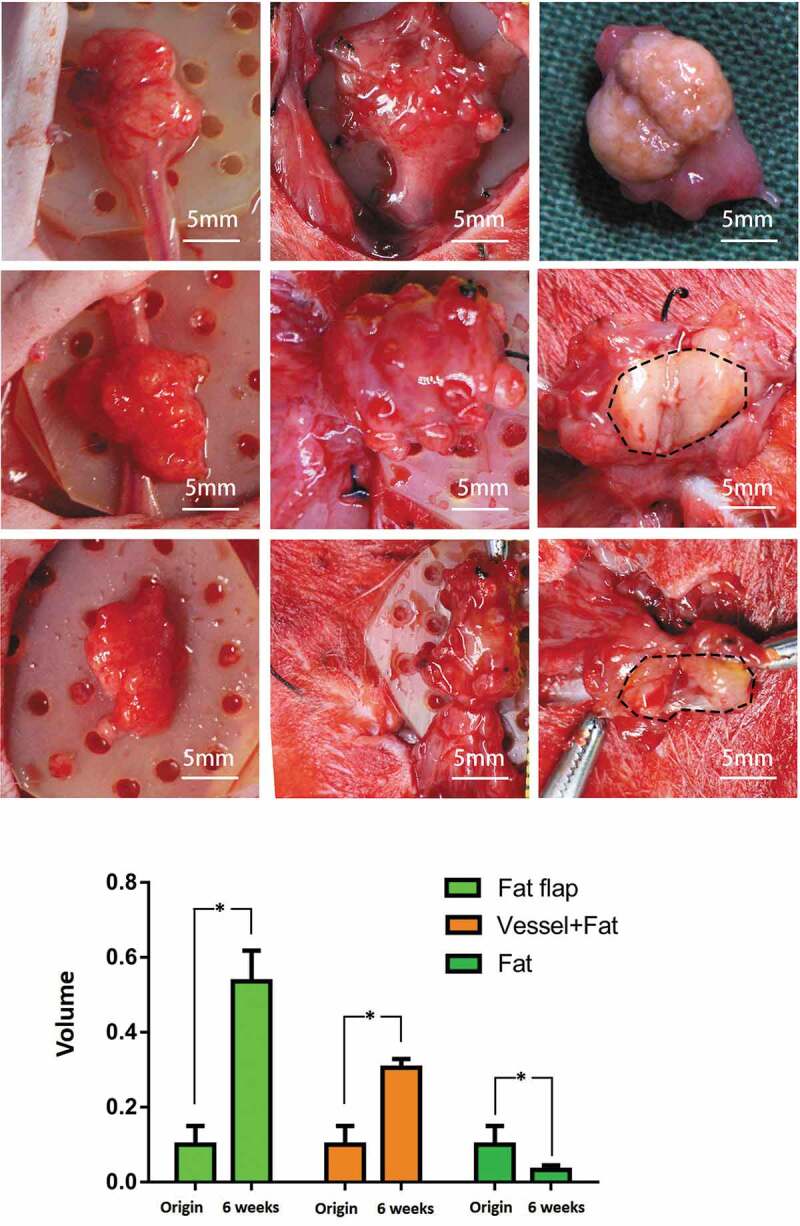
The macroscopic view of adipose tissue and volume change in three group. Equal volume of adipose tissue was set as the initial volume. At week 6, the first line: fat flap enlarged and the crossing section view showed the adipose tissue appearance. The borders between host tissue and neo-tissue was marked with dotted line. The second line: The volume of grafted fat increased significantly (P < 0.05) and grew with the ligated vessel as a whole. The third line: The volume of grafted fat alone decreased, the wrapping fibrous tissue seemed to be thick. The volume of construct increased significantly in group A (P < 0.05) and group B (P < 0.05) while the volume in group C decreased significantly (P < 0.05)

In group A (fat flap group): The appearance of the tissue looked fresh, the texture of the fibrous tissue felt soft when we used forceps to elevate the flap, and the axial blood flow was well-functional as shown in Figure 2. The micro-vessels were visible around the tissue. The volume of the tissue in the engineering chamber was significantly increased to 0.536 ± 0.082 ml (P < 0.05) from 0.1 ml as shown in Figure 2. At week 6, the volume in group A was significantly higher than that of in group B and C with both P < 0.05. The crosssing observation in Figure 2 showed no obvious border of neo-tissue and the original flap.

In group B (grafted fat with ligated vessels): The tissue had a similar appearance to the tissue in group A. When being poked, the capsule felt tougher than that of in group A. The axial blood flow was normal according to the clamping test. The volume increased to 0.306 ± 0.023 ml from 0.1 ml, significantly higher than the original volume (P < 0.05) and significant higher than that of in group C at week 6 (P < 0.05). When observed from the crossing-section view, there was a clear border between the adipose tissue and fibrous tissue in Figure 2.

In Group C (grafted fat without vessel): the tissue was hard and irregular, the capsule was thick. The volume significantly reduced to 0.033 ± 0.012 ml (P < 0.05).

### Microscopic observation and composition analysis

**Figure 3.**
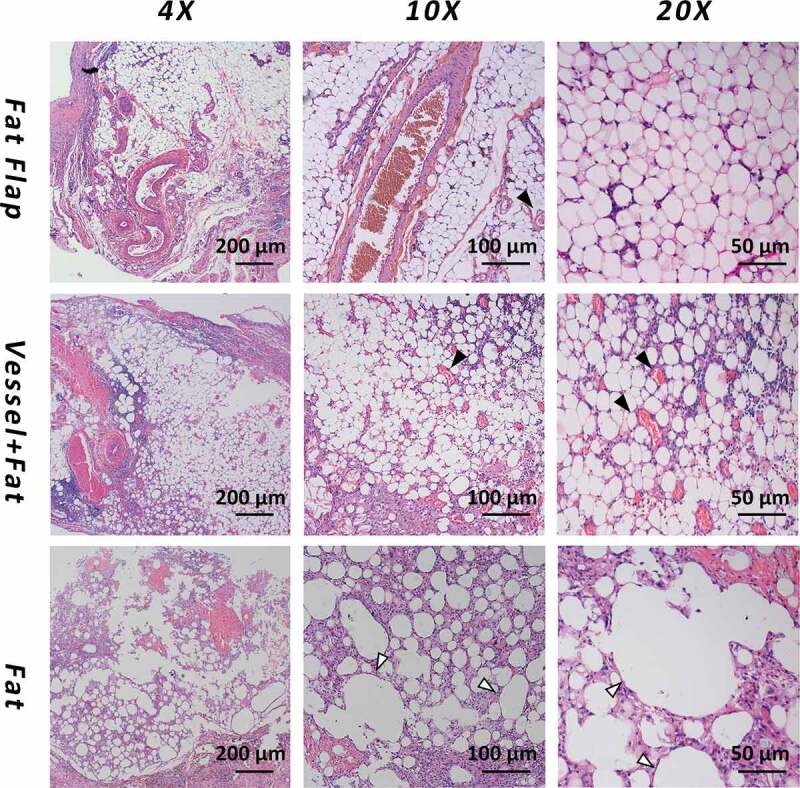
The H&E staining of adipose tissue in each group at week 6. The first line: adipocytes were homogenous, distributed around the axial blood vessels with a small number of inflammatory cells infiltrating. The second line: Most of the adipocytes showed normal morphology. A large amount of blood vessels within the generated stromal matrix could be observed with erythrocytes passing through showing a sign of functional blood vessels. The third line: The majority of tissue in group C was necrotic adipose tissue and it was wrapped in a thick capsule. Vessels were marked as black arrow and the necrotic droplet was marked as the white arrow

**Figure 4.**
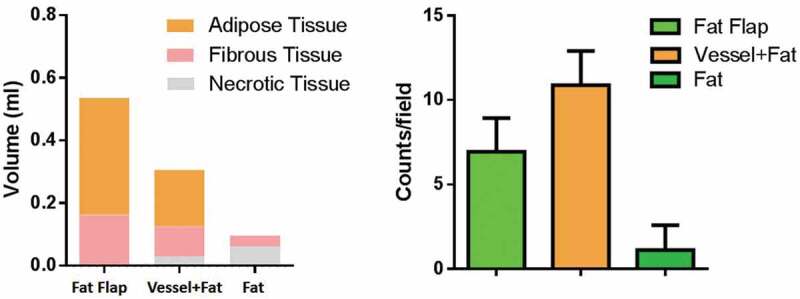
The composition of construct analysis and vessel counts quantification. The construct in fap flap group and vessel+Fat group majorly consisted of adipose tissue. Necrotic tissue was the main component in fat alone group. The cell counts result showed that the vessels in the vessel+fat group were significantly higher than that of in the fat flap group and fat alone group (P < 0.05)

After 6 weeks, the tissue in the chamber contained two kinds of tissues as shown in Figure 3: the capsule composed of fibrous-like tissue and the adipose tissue was wrapped by the capsule. These two kinds of different tissues were easy to distinguish from each other. There was also necrotic tissue in group B and group C. The composition ratio figure could be found in Figure 4 and the exact number was listed in the supplemental Table 1.

In group A (fat flap group): adipocytes were homogenous, distributed around the axial blood vessels with a small number of inflammatory cells infiltrating in Figure 3. The appearance was similar to normal adipose tissue. The estimated volume of adipose tissue was 0.373 ± 0.045 ml (67.4%), while the volume of connective tissue was 0.174 ± 0.043 ml (31.6%)

In group B (grafted fat with ligated vessels): Most of the adipocytes showed normal morphology. A large amount of blood vessels within the generated stromal matrix could be observed with erythrocytes passing through showing a sign of functional blood vessels (Figure 3, black arrow). The vessels counts showed a higher number of vessels in group B than that of in group A. (P < 0.05). . The estimated volume of adipose tissue was 0.163 ± 0.032 ml (55.8%), while the connective tissue was 0.096 ± 0.038 ml (33.0%)

A small amount of necrosis (0.033 ± 0.045 ml, 11.2%) could be observed.

In group C (grafted fat without vessel): The majority of tissue in group C was necrotic adipose tissue (0.062 ± 0.018 ml, 66.5%) and it was wrapped in a thick capsule. There was rare vascular structure while large droplet vacuole could be observed (Figure 3, white arrow).

### Micro CT vessel reconstruction

**Figure 5.**
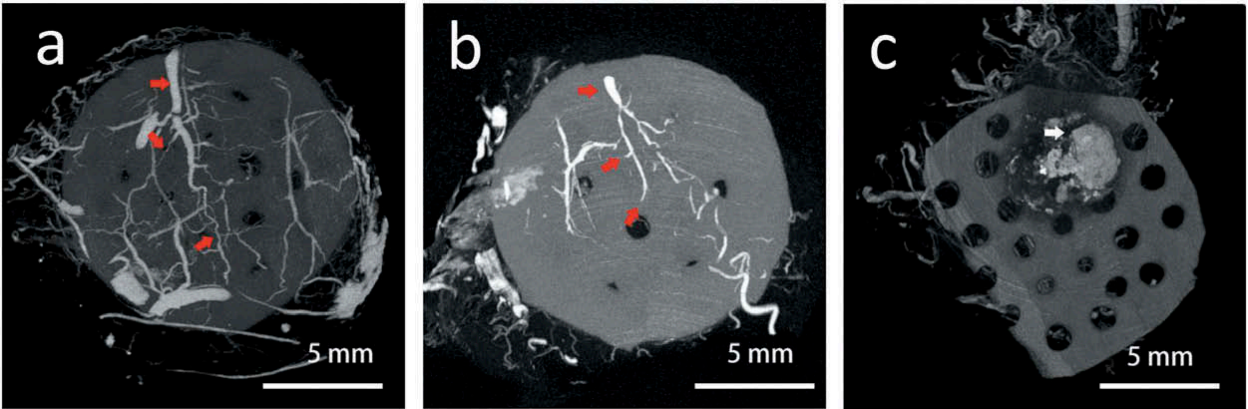
The reconstruction of vessel structure by micro CT scan. (a) the whole tissue was well-vascularized. The vessel pedicle within the chamber could be seen clearly as the right arrow showed. (b) There was also a clear pedicle vessel with several branches, but less branches could be observed than that of in group A. (c) there was no obvious vascular structure within or around the grafted fat, massive calcification could be seen as the necrotic sign

After perfusion of the angiographic contrast agent. The vascular structure could be seen wrapping around the tissue engineering chamber. The vascular reconstructive image in group A showed that the whole tissue was well vascularized. The vessel pedicle within the chamber could be seen clearly (Figure 5(a), red arrow). Branches from the axial artery connected with the vessels derived from the surrounding tissue. In group B: There was also a clear pedicle vessel with several branches, but less branches could be observed than that of in group A. In the group C, There was no obvious vascular structure within or around the grafted fat, massive calcification could be seen as the necrotic sign (Figure 5(c), white arrow).

## Discussion

Fat grafting has become more and more popular in the last decade [21]. Early revascularization is thought to be crucial for the retention rate and most of the tissue volume was kept by the regenerated adipose tissue instead of surviving according to the theory of Yoshimura [3]. Besides this, CAL is based on the mechanism that adipose-derived stromal/stem cells could promote angiogenesis via angiogenic growth factor paracrine instead of differentiating into cell components [22–27]. Although the differential potential of ASCs was still thought to be promising, there was no doubt that revascularization is essential in determining the result of fat graft and there were plenty of studies proved that angiogenic growth factor could also promote the retention of grafted fat [28,29].

Tissue engineering chamber model (TEC) was a newly developed model that has shown great potential in tissue engineering by its vascular supporting of engineered tissue [14,17]. By introducing bundles of ligated vessels, this model can promote the survival and proliferation of even myocardial cells [14]. Furthermore, when introducing tissue with the intact vascular system, a number of studies have shown their results of enlarging the volume of fat flaps in rats [15], rabbits [16] and pigs [17] in TEC. This kind of spontaneous regeneration seemed to be specific in adipose tissue. But when TEC was applied in clinic later, the result seemed to be unpredictable [20] and there was no further large-scale clinical experiment so far.

Our study demonstrated that when inducing a bundle of ligated vessels to grafted fat and there was robust angiogenesis derived from the induced vessels that can support the survival and retention of grafted fat according to the volume change and histological findings (Figure 2). The vessel counts were coordinate with this point (Figure 4), while the angiography result of micro CT showed less branches in group B compared with that of in group A that was set as positive control. One of the reasonable explanations was that the remodelling process of grafted fat in group B didn’t complete as there were still inflammatory cell infiltrating (Figure 2) and as a process of angiogenesis, after sprouting and branching, with the maturation of blood vessels, it will be vascular remodelling and finally regression [30]. This phenomenon was also proposed in the previous study in TEC model [31]. As the neocapillary may not be perfused by the angiography contrast agent, there were less branches could be seen in the angiography.

But it was obvious from the reconstructive image that the grafted fat was nourished by the induced vessel. On the other hand, the grafted fat without the vessel showed massive necrosis and calcification (Figure 5, white arrow). Therefore our construct could be treated as a fat flap. Interestingly, the volume of grafted fat in group B increased with the vessel, According to the previous study, there was rare reports on lipohyperplasia after fat grafting. Here our result suggests while grafted fat could be well-revascularized under a favoured environment like TEC, the grafted fat could not only preserve but also regenerate. Although the initial volume of our study was small (0.1 ml), we used bolus fat transplantation and the grafted fat in group C, the negative control group, showed necrosis. Our results could not reflect the upper limit of bolus transplantation with blood vessels. Large animal studies could be conducted to explore the potential of this model.

As mentioned above, total breast reconstruction with fat graft will require a high number of procedures [10,32]. Therefore, the efficiency of fat grafts during breast reconstruction was important. Although radiotherapy is the most important factor associated with the number of treatment sessions according to a meta-analysis [33]. Limited layer for transplantation was an obvious obstacle even muscular and submuscular layers were considered as target layers [34]. According to present clinical principle, grafted fat should be scattered [21,35] and bolus fat transplantation should be avoided [12]. But this principle seemed to be hard to achieve perfectly with limited injection layer option. In fact, there was no method present to certificate the grafted fat was scattered. Our study showed a potential of induction of vessel during fat grafting, which may create a new ‘layer’ of adipose tissue and for further fat grafting and may utilize the space that was used to be poor recipient site after the removal of the expander [36,37]. The strategy may also contribute a satisfying aesthetic result because it could be similar to a prosthesis but with natural texture from the filling view. Furthermore, the cell-assisted lipotransfer also araised the concern of oncological safety [36,38] while our strategy only provides a ligated vessel that decreases the risk.

There was obvious limitations of our present study, we used a silicone chamber that was implanted as a foreign material. But considering this was a primary study, next step we will try a further experiment in large animal to testify the potential of the bolus fat grafting with ligated vessels and without chamber implantation and thus we can make this strategy stable by achieving a consistent retention rate of grafted fat.

In conclusion, fat grafts in tissue engineering chamber model could survive and maintain well. The combination of fat graft and tissue engineering chamber could fabricate a vascularized fat flap. Well-vascularized fat grafts may even be enlarged by tissue engineering chamber technique. The revascularization degree of grafted fats determined the result of transplantation. Different methods besides cell therapy that cloud promote the vascularization could be considered to improve the transplantation procedure.
